# Modern Tendencies in Hospital Administration

**Published:** 1915-12-11

**Authors:** 


### KING EDWARD'S HOSPITAL FUND.

### Modern Tendencies in Hospital Administration.

1915 Statistics (Third Notice).

Protagonists of the movement for the adoption of a Uniform System of Accounts for hospitals, institutions, and other charities had always in mind the propriety of disclosing in the annual accounts and reports all information essential to a member of the public who desires to study the conditions of management at individual institutions with a view to dividing the amount of money he feels able to distribute in the form of charity.

In respect of one matter in particular, such a prospective donor most often fails to find in reports certain information which we have always urged should invariably accompany any statement as to the year's working; we allude to particulars as to the size of the staff—medical, nursing, dispensing, secretarial, and general, information which enables one readily to compute the extent of a condition which has an important bearing upon almost every heading on the expenditure side of the income and expenditure account: the number of mouths to feed, the number of persons to clothe in uniform, the extent of the beds and the tenders of the beds for whom heating, light, and housing have to be provided; the extent of the laundry arrangements for such a number, the renewal and repair of furniture, the provision of bedding and linen, crockery, and other things requisite to their comfort.

### Fuller Information Needed.

Consistently, from its earliest edition in 1893, Burdett's "Uniform System of Accounts" has urged the publication of these particulars. Originally in this work a form of account was suggested in which to enter separately the expenditure upon the maintenance of the nursing staff in each hospital or institution. This had the double utility of enabling anyone who examined the accounts to form an opinion as to whether this branch of the staff was adequate or inadequate to the size of the institution, and at the same time reminded the public that the number of persons to provide for was necessarily largely in excess of the officially stated number of beds for patients. The text-book referred to, moreover, urged "the authorities of every hospital and institution, which aims at securing a reputation for sound and economical administration, to publish in the report, in conjunction with the income and expenditure account, a table . . . of persons boarded and partly boarded."

### Modern Tendencies.

Of course, twenty or thirty years ago the nursing section of the staff of a hospital accounted for a very large proportion of those whom it was found necessary to employ to carry on the work. Nowadays the proportion is nothing like so great, although the number of nurses to each bed is probably more and their training for their work has been elaborated. The size of the staff in other departments has increased, and entirely new classes of workers in different spheres, who in former times were not even thought of, are now a common feature in the properly equipped hospital of to-day. Let us examine in what way these conditions, inherent to the scientific advance of the art of healing, have increased the responsibilities of those whose duty it is to gather in the wherewithal to provide for the needs of the patients and the staff. We have said that nurses' training has been elaborated. The keen woman who seriously takes up nursing as a career very properly and wisely wishes to enter the school where she will learn most and not necessarily earn most. It follows that teachers must be provided, otherwise an earnest and desirable class of probationers will not be forthcoming. In each subject—in midwifery, massage, the application of medical electricity, physical exercises, general nursing—there must be a professor; and even if a regular member of the nursing staff, such as a matron or assistant matron, is the teacher, expenditure on salaries and extent of staff are both augmented, for obviously the duties previously performed by these officers cannot without assistance be carried out in addition to the function of giving instruction.

On the medical side we find also an increase in numbers to meet the requirements of modern times, embracing extensive notetaking, more complete clinical examination of the patient, the injection of serums, etc., and the many other duties which would readily suggest themselves to a medical man were he dealing with this side of the subject.

In the pharmacists' department there is a larger number of workers occasioned by the requirements of a more exacting medical profession and by the common knowledge that it is more economical to manufacture or prepare some forms of medicine within the walls of the hospital than to buy them in their final state from without. The *x*-ray department is an innovation of comparatively modern times The pathological section, to keep pace with the work of the physicians and surgeons, claims more skilled workers. The theatre is a totally different thing from the operating room of past times and needs its special staff. In these and other ways a large proportion of miscellaneous workers, extra to what was once the main factor of the general staff, are added to the number of "mouths to feed."

### Salaries and Wages.

And in what way can the student of the average hospital report or of the Statistical Return of the King's Fund gain any knowledge of the extent to which these conditions prevail in the individual hospital or in the group of hospitals devoted to general or specialised work? By one means only, and that by examining and comparing the expenditure under salaries and wages, conscious while he is doing so that there are no standard rates of remuneration, and that notoriously payment differs widely amongst institutions and is often made, in all branches, partly in cash, partly in instruction, and partly in opportunities of advancement.

The matter is important both from a statistical point of view and in reference to the opportunity that should be given to an interested inquirer to determine the extent to which an institution is or is not properly equipped with a suitable staff of workers, or is, as may easily be, overstaffed. We claim indulgence. therefore, to quote from a previous article on this subject:

"To speak of a hospital having 150 occupied beds means that the daily average of beds occupied is 150, that there are 150 people to feed, to attend medically, to nurse, to wash for, to tend generally every day. The statement gives no indication of the number of other inmates of the institution who, in addition, require several of these services. These may number fifty, possibly one hundred; the extent of the establishment is, at present, left entirely to the imagination or knowledge of the reader of the Statistical Report."

A Contrast.

In dealing with this a ready means of expression finds itself in the form of figures. We take two hospitals each doing precisely the same class of work, and each having the same daily average of occupied beds. The total number of inmates is as follows:—

| | Hospital "A" | Hospital "B" |
|---|---|---|
| Patients ... ... ... ... | 150 | 150 |
| Staff : | | |
| *Medical.*—Pathologist ... ... | 1 | 1 |
| Registrar ... ... ... ... | 1 | 0 |
| House Officers ... ... ... | 3 | 2 |
| *Dispensary.*—Dispensers ... ... | 3 | 2 |
| Porter or boy ... ... ... | 1 | 1 |
| *Nursing.*—Matron ... ... ... | 1 | 1 |
| Assistant Matron or Home Sister | 1 | 1 |
| Housekeeper ... ... ... | 1 | 0 |
| Night Sister ... ... ... | 1 | 1 |
| Theatre Sister ... ... ... | 1 | 1 |
| Ward Sisters ... ... ... | 7 | 6 |
| Electrical or Out-patient Sister | 1 | 1 |
| Massage Sister ... ... ... | 1 | 0 |
| Nurses ... ... ... ... | 52 | 36 |
| *Engineers and Stokers* ... ... | 4 | 2 |
| *Porters, Carpenters, etc.* ... ... | 8 | 6 |
| *Secretary's Department* ... ... | 5 | 3 |
| *Steward* ... ... ... ... | 0 | 1 |
| Totals ... ... ... | 242 | 215 |
| Deduct non-resident, therefore counted as half full board ... | (10) 5 | (8) 4 |
| | 237 | 211 |
| Deduct number allocated to out-patient department ... ... | 4 | 4 |
| Total for purposes of financial calculation ... ... ... ... | 233 | 207 |

| | | £ | s. | d. |
|---|---|---|---|---|
| Annual cost per bed occupied by a patient (including salaries, wages, and board of staff) ... ... ... | "A" | 84 | 18 | 8 |
| | "B" | 76 | 13 | 4 |
| Annual average cost of each "mouth to feed" (including patients and workers resident or partly boarded) | "A" | 54 | 13 | 6 |
| | "B" | 55 | 11 | 1 |

We admit that the table we have provided is capable of improvement, but in the form suggested or one closely resembling it, should the hospitals generally agree to present the information, the prospective benefactor would have a far ampler opportunity of coming to a decision as to where to bestow his alms. He would, in addition, be better able to discover where the work is being done "on the cheap," and to appraise the value of the teaching machinery of each institution. If he still feels that the cost per occupied bed worked out on the old lines should be the proper gauge of economy, the figure will remain at his service.

The latest issue of the King's Fund Statistical Tables reminds us that this may be the time for the parting of the ways in methods of the presentation of hospital statistics, and an opportunity for considering new or improved forms of preparing figures. This being so, we ask hospital administrators, who represent the greatest influence in the matter, carefully to consider the suggestions we have ventured to make, and to decide if these ideas, not new or unsupported by the opinion of capable thinkers, may not in some way be incorporated in forthcoming alterations and improvements.

